# AICAR ameliorates high-fat diet-associated pathophysiology in mouse and ex vivo models, independent of adiponectin

**DOI:** 10.1007/s00125-017-4211-9

**Published:** 2017-02-10

**Authors:** Emma Börgeson, Ville Wallenius, Gulam H. Syed, Manjula Darshi, Juan Lantero Rodriguez, Christina Biörserud, Malin Ragnmark Ek, Per Björklund, Marianne Quiding-Järbrink, Lars Fändriks, Catherine Godson, Kumar Sharma

**Affiliations:** 1 The Wallenberg Laboratory for Cardiovascular and Metabolic Research, Institute of Medicine, Sahlgrenska Academy, University of Gothenburg, Bruna Stråket 16, S-413 45 Gothenburg, Sweden; 2 Centre for Renal Translational Medicine, Institute of Metabolomic Medicine, UC San Diego Health Sciences, San Diego VA HealthCare System, Stein Clinical Research Building, Room 406, mail code 0711, 9500 Gilman Drive, La Jolla, CA 92093 USA; 3 Veteran’s Affairs (VA), San Diego VA HealthCare System, Veterans Medical Research Foundation, San Diego, CA USA; 4 Department of Gastrosurgical Research and Education, Institute of Clinical Sciences, Sahlgrenska Academy, University of Gothenburg, Gothenburg, Sweden; 5 Division of Infectious Diseases, School of Medicine, University of California, San Diego, CA USA; 6 Department of Microbiology and Immunology, Institute of Biomedicine, Sahlgrenska Academy, University of Gothenburg, Gothenburg, Sweden; 7 University College Dublin (UCD) Diabetes Complications Research Centre, UCD Conway Institute, School of Medicine and Medical Sciences, University College Dublin, Dublin, Ireland

**Keywords:** Adiponectin, AICAR, Inflammation, Kidney disease, Liver disease, Macrophages, Obesity

## Abstract

**Aims/hypothesis:**

In this study, we aimed to evaluate the therapeutic potential of 5-aminoimidazole-4-carboxamide ribonucleotide (AICAR), an activator of AMP-activated protein kinase, for ameliorating high-fat diet (HFD)-induced pathophysiology in mice. We also aimed to determine whether the beneficial effects of AICAR were dependent on adiponectin. Furthermore, human adipose tissue was used to examine the effect of AICAR ex vivo.

**Methods:**

Six-week-old male C57BL/6J wild-type and *Adipoq* ^−/−^ mice were fed a standard-fat diet (10% fat) or an HFD (60% fat) for 12 weeks and given vehicle or AICAR (500 μg/g) three times/week from weeks 4–12. Diet-induced pathophysiology was examined in mice after 11 weeks by IPGTT and after 12 weeks by flow cytometry and western blotting. Human adipose tissue biopsies from obese (BMI 35–50 kg/m^2^) individuals were incubated with vehicle or AICAR (1 mmol/l) for 6 h at 37°C, after which inflammation was characterised by ELISA (TNF-α) and flow cytometry.

**Results:**

AICAR attenuated adipose inflammation in mice fed an HFD, promoting an M1-to-M2 macrophage phenotype switch, while reducing infiltration of CD8^+^ T cells. AICAR treatment of mice fed an HFD partially restored glucose tolerance and attenuated hepatic steatosis and kidney disease, as evidenced by reduced albuminuria (*p* < 0.05), urinary H_2_O_2_ (*p* < 0.05) and renal superoxide levels (*p* < 0.01) in both wild-type and *Adipoq* ^−/−^ mice. AICAR-mediated protection occurred independently of adiponectin, as similar protection was observed in wild-type and *Adipoq* ^−/−^ mice. In addition, AICAR promoted an M1-to-M2 macrophage phenotype switch and reduced TNF-α production in tissue explants from obese human patients.

**Conclusions/interpretation:**

AICAR may promote metabolic health and protect against obesity-induced systemic diseases in an adiponectin-independent manner. Furthermore, AICAR reduced inflammation in human adipose tissue explants, suggesting by proof-of-principle that the drug may reduce obesity-induced complications in humans.

***Trial registration*:**

ClinicalTrials.gov NCT02322073

**Electronic supplementary material:**

The online version of this article (doi:10.1007/s00125-017-4211-9) contains peer-reviewed but unedited supplementary material, which is available to authorised users.

## Introduction

The obesity pandemic poses a serious public health challenge. Obesity is associated with numerous pathologies, including diabetes, non-alcoholic fatty liver disease and kidney disease [1, 2]. There are currently more overweight than underweight people worldwide [3]; this increase in prevalence has prompted a search for effective treatments to tackle obesity-related pathophysiology.

The adenosine monophosphate analogue 5-aminoimidazole-4-carboxamide ribonucleotide (AICAR) activates AMP-dependent protein kinase (AMPK), which is a key regulator of energy metabolism and thus a potential target for treatment of obesity-related complications [4, 5]. Evidence from experimental models has shown that AICAR may attenuate metabolic [6–8], hepatic [9–11] and renal pathophysiology [12–15]. However, the use of AICAR could be compromised as some reports indicate that AICAR increases adiponectin production [13]. Although adiponectin is generally described as a protective adipokine [16], several clinical studies have reported a paradoxical inverse association between circulating adiponectin levels and renal function [17, 18]. Specifically, increased adiponectin levels correlate with diabetic nephropathy [19, 20], advanced chronic kidney disease (CKD) [21–23] and increased risk of mortality [24]. As we cannot exclude the compromising role of adiponectin in CKD patient groups, it is critical to assess if AICAR-mediated actions are adiponectin dependent to determine its suitability as a drug to target obesity-related pathophysiology.

The aims of this study were to evaluate the therapeutic potential of AICAR for the promotion of metabolic health and reduction of liver and kidney disease in mice fed a high-fat diet (HFD) and to determine if such protection was dependent on adiponectin. Since white adipose tissue (WAT) inflammation is a key driver of obesity-related pathophysiology [25–29], this was characterised in detail. Finally, to translate our rodent data to human pathophysiology, we also investigated whether AICAR could reduce inflammation in omental WAT tissue explants obtained from obese individuals undergoing gastric bypass surgery.

## Methods

### Animal study

Six-week-old male wild-type (C57BL/6J) and *Adipoq* ^−/−^ (B6;129-*Adipoq* ^tm1Chan^/J) mice (catalogue numbers 000664 and 008195, respectively; The Jackson Laboratory, Sacramento, CA, USA) were housed in a temperature/humidity controlled room on a 12 h light/12 h dark cycle. Mice were allowed to acclimatise for a minimum of one week prior to commencement of experiments, as described in the electronic supplementary material (ESM) Methods. Mice were fed a sucrose-matched standard-fat diet (SFD; 10% fat) or an HFD (60% fat) for 12 weeks (*n* = 3–17 per experiment, as indicated in the respective figure legends). During weeks 4–12 of SFD/HFD feeding, vehicle or AICAR (500 μg/g [13]) was given three times per week via i.p. injections. Mice were randomly assigned in consecutive order to an SFD or HFD, and to vehicle or AICAR treatment. At week 11, we performed a fasted IPGTT [30] and collected urine for 24 h to assess microalbuminuria and urine H_2_O_2_ levels. At the end of the study, WAT and kidney leucocytes were isolated and characterised by flow cytometry. AMPK activation in WAT was determined by western blot analysis. Liver morphology was visualised by haematoxylin and eosin staining and hepatic free cholesterol and triacylglycerol concentrations were measured using standardised kits (cholesterol: WAKO, Richmond, VA, USA; triacylglycerol: Pointe Scientific, Canton, MI, USA). Plasma creatinine was measured by HPLC. In a subset of mice, dihydroethidium (DHE, 50 mg/kg) was administered by i.p. injection 16 h before the mice were killed to quantify renal superoxide levels by confocal imaging (*n* = 4 per group) [14]. Detailed protocols for liver function analysis, HPLC plasma creatinine analysis and renal superoxide measurements are described in ESM Methods.

### J774 experiments

Serum-starved J774 macrophages were treated with vehicle or AICAR (1 mmol/l) for 16 h [31]. Cellular p-AMPK/AMPK was quantified by western blot analysis. Cells were characterised by flow cytometry as proinflammatory M1 macrophages (CD11c^+^) or anti-inflammatory M2 macrophages (CD206^+^); values from vehicle-treated cells were set at 100%.

### Human adipose explant culture

Omental WAT explants were obtained from obese (BMI 35–50 kg/m^2^) non-diabetic individuals (*n* = 4) undergoing bariatric surgery. WAT explants were incubated ex vivo (1 g tissue per 2 ml DMEM) with vehicle or AICAR (1 mmol/l) for 6 h at 37°C (see ESM Methods for further details). Supernatant TNF-α levels were determined by ELISA and tissue leucocytes were characterised by flow cytometry.

### Leucocyte phenotyping by flow cytometry

Tissue leucocytes were isolated by treating WAT and kidneys with collagenase (5 mg/ml and 10 mg/ml, respectively) at 37°C [29, 32]. Lysates were filtered (70 μm pore size) and 5 × 10^5^ cells were stained by Aqua-Live-Dead (Thermo Fisher, Waltham, MA, USA) and relevant antibodies for characterisation (see ESM Table 1 and ESM Table 2 for antibody details). Both human and murine lymphocytes (CD3^+^CD45^+^) were characterised as T helper (% CD4^+^ of CD3^+^CD45^+^) and cytotoxic T cells (% CD8^+^ of CD3^+^CD45^+^). In murine tissues, F4/80^+^ macrophages (CD45^+^F480^+^) were subcategorised as inflammatory M1 macrophages (% CD11c^+^ of CD45^+^F480^+^) or M2 macrophages (% CD206^+^ of CD45^+^F480^+^). In human WAT, the mononuclear/macrophage population (CD45^+^) was identified as M1 macrophages (% CD11c^+^ of CD45^+^), M2a macrophages (% CD206^+^ of CD45^+^), M1/M2b macrophages (% CD86^+^ of CD45^+^) or M2a/M2c macrophages (% CD163^+^ of CD45^+^). Gating was determined using Fluorescence-Minus-One controls (see ESM Methods for further details).

### Western blot analysis

Adipose tissue and serum-starved J774 macrophages were homogenised in RIPA lysis buffer. Briefly, 40 μg protein was loaded onto a 16% SDS-PAGE gel and transferred onto polyvinylidene difluoride (PVDF) membranes (0.2 μm pore size) [31]. Proteins were identified using rabbit anti-p-AMPKα (Thr172; no. 2535s), rabbit anti-AMPKα (no. 2532s) and rabbit anti-adiponectin (no. 2789) antibodies (all diluted 1:1000; Cell Signaling, Danvers, MA, USA). Proteins were normalised against β-actin (mouse anti-β-actin antibody [no. A2228]; Sigma, St Louis, MO, USA), diluted 1:10,000 (see ESM Methods for further details). Original western blot images were cropped as indicated by vertical lines.

### Study approval

The Veterans Affairs San Diego Healthcare System Institutional Animal Care and Use Committee (IACUC) approved all animal procedures (approval no. 10-029), and the *Guide for the Care and Use of Laboratory Animals* was followed during experiments. Human tissue specimens were obtained from a larger study (ClinicalTrials.gov NCT02322073) in agreement with the principal investigator, V. Wallenius. The Regional Ethical Review Board (Gothenburg, Sweden) approved all study procedures (Dnr 682-14) and all patients were enrolled in accordance with the Helsinki Declaration. Written informed consent was obtained from all participants included in this study.

### Statistical analyses

Gaussian distribution was assumed and two-tailed Student’s *t* test, or two-way ANOVA with paired Bonferroni correction as a post hoc comparison was used, as indicated in the figure legends. Analyses were performed using GraphPad Prism version 5 (La Jolla, CA, USA), licensed to UC San Diego.

## Results

### AICAR attenuated HFD-induced adipose inflammation independent of adiponectin

Wild-type and *Adipoq* ^−/−^ mice were fed a sucrose-matched SFD (10% fat) or HFD (60% fat) for 12 weeks (ESM Fig. 1a). Because this HFD regime has previously been shown to cause systemic disease after 4 weeks, such as renal impairment [13], we initiated AICAR treatment at week 4 to test the effect of AICAR as an intervention.

As expected, HFD-fed mice gained significantly more weight than mice fed an SFD (ESM Fig. 1b). AICAR is known to increase metabolism and weight loss [7], even in sedentary mice [8]. Accordingly, we observed that AICAR attenuated weight gain during the final few weeks of the diet regimen, both in wild-type and *Adipoq* ^-/-^ mice (ESM Fig. 1b). However, AICAR-treated HFD-fed mice weighed significantly more than controls fed an SFD in both mouse strains throughout the study (ESM Fig.1b).

**Fig. 1:**
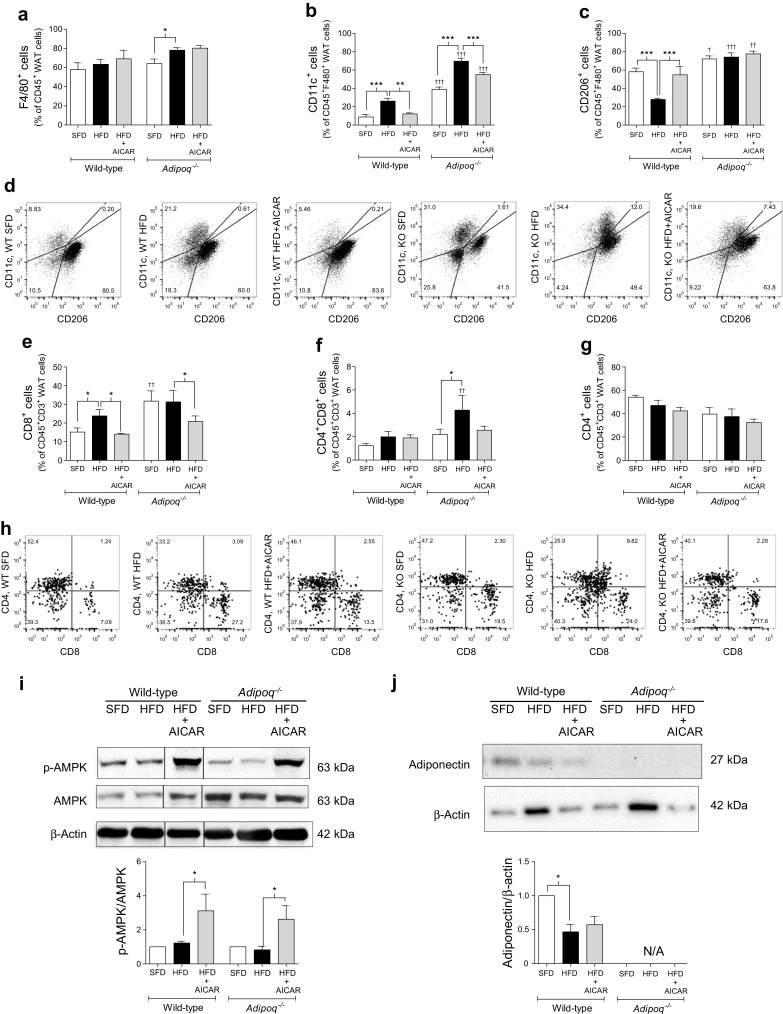
AICAR attenuates adipose inflammation independent of adiponectin in obese mice. Wild-type and *Adipoq* ^−/−^ mice fed a 12 week SFD (10% fat) or HFD (60% fat) received vehicle or AICAR (500 μg/g) during weeks 4–12. WAT leucocytes were characterised by flow cytometry; (**a**)–(**d**) Macrophages were characterised as pan-macrophages (F4/80^+^), proinflammatory M1-macrophages (CD11c^+^) or anti-inflammatory M2-macrophages (CD206^+^) (*n* = 5). (**e**)–(**h**) Lymphocytes were characterised as T killer (CD8^+^) vs T helper (CD4^+^) cells (*n* = 5). (**i**), (**j**) AMPK activation and adiponectin levels were analysed by western blot. AMPK blots were cut as indicated (full blots and details of cutting are presented in ESM Fig. 4) (*n* = 3). Data are presented as mean ± SEM. *p < 0.05, **p < 0.01, ***p < 0.001; ANOVA with Bonferroni correction; ^†^ *p* < 0.05, ^††^ *p* < 0.01, ^†††^ *p* < 0.001, *Adipoq* ^−/−^ vs wild-type mice for respective conditions. N/A, not applicable

The total number of F4/80^+^ macrophages was not affected by HFD in perigonadal WAT from wild-type mice (Fig. 1a), in accordance with our previous studies [29]. However, HFD-fed *Adipoq* ^−/−^ mice presented with an increased number of F4/80^+^ macrophages (*p* < 0.05; Fig. 1a). *Adipoq* ^−/−^ mice fed either diet exhibited a higher percentage of CD11c^+^ M1 macrophages compared with their respective wild-type controls (*p* < 0.001; Fig. 1b).

AICAR attenuated HFD-induced WAT inflammation in both mouse strains; in wild-type mice, AICAR treatment reduced the percentage of CD11c^+^ M1 macrophages (*p* < 0.01) and increased levels of anti-inflammatory CD206^+^ M2 macrophages (*p* < 0.001; Fig. 1b–d). Similarly, AICAR attenuated HFD-induced CD11c^+^ M1 macrophages in *Adipoq* ^−/−^ mice (*p* < 0.001). Macrophage CD206^+^ expression was increased in SFD-fed *Adipoq* ^−/−^ mice compared with SFD-fed wild-type mice, but there were no changes between *Adipoq* ^−/−^ mice on an SFD or an HFD with or without AICAR treatment (Fig. 1c). AICAR also reduced the percentage of cytotoxic CD8^+^ T cells in both mouse strains but did not affect levels of CD4^+^ T cells (Fig. 1e–h). Although we and others have previously shown that HFD reduces p-AMPK/AMPK [13], surprisingly HFD did not alter AMPK activity in WAT in the present study. This may be explained by the fact that we matched sucrose levels in SFD and HFD regimens in the present study. However, as expected, AICAR increased WAT p-AMPK/AMPK levels (Fig. 1i). Furthermore, AICAR did not restore HFD-mediated attenuation of WAT adiponectin in wild-type mice (Fig. 1j).

**Fig. 2:**
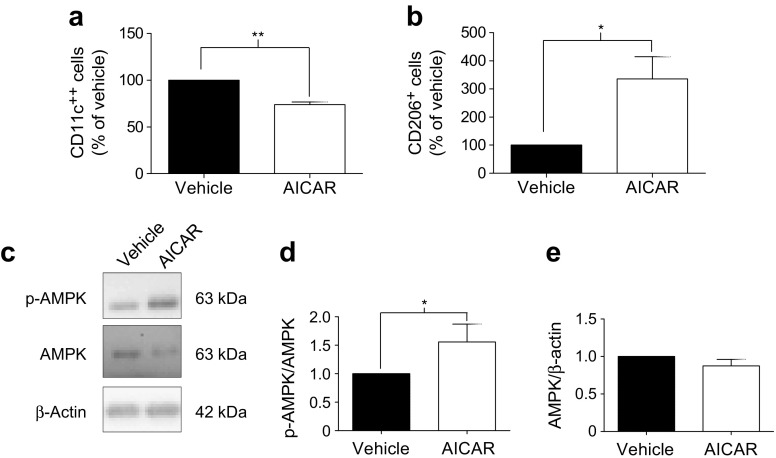
AICAR promotes AMPK activation and an M1-to-M2 phenotype switch in cultured macrophages. J774 macrophages were incubated with vehicle or AICAR (1 mmol/l) for 16 h (*n* = 3). Cells were characterised as (**a**) proinflammatory M1 (CD11c^++^) or (**b**) anti-inflammatory M2 (CD206^+^) by flow cytometry and (**c**–**e**) p-AMPK/AMPK was assessed by western blot. Data are presented as mean ± SEM. *p < 0.05, **p < 0.01, paired Student’s *t* test

WAT comprises a myriad of cells, including adipocytes, epithelial cells and leucocytes. To determine if AICAR could alter the macrophage phenotype via direct or indirect effects, we also investigated AICAR-mediated effects on murine macrophages in vitro, using the J774 cell line. Similar to the in vivo findings, AICAR promoted an M1-to-M2 phenotype switch in cultured macrophages, attenuating CD11c^++^ expression (*p* < 0.01), while promoting CD206^+^ expression (*p* < 0.05; Fig. 2a,b). This correlated with increased p-AMPK/AMPK in this cell line (*p* < 0.05; Fig. 2c,d), but AMPK/β-actin remained unaltered (Fig. 2c,e).

### AICAR partially restored glucose tolerance in obese mice independent of adiponectin

**Fig. 3:**
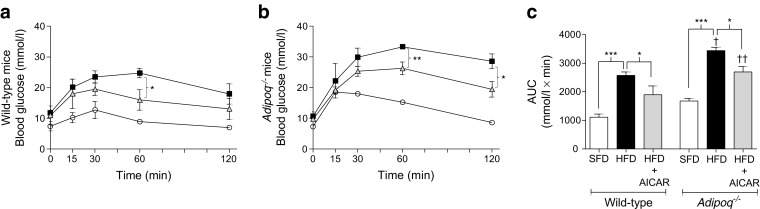
AICAR partially restores glucose tolerance independent of adiponectin in obese mice. Wild-type and *Adipoq* ^−/−^ mice fed a 12 week SFD (10% fat) or HFD (60% fat) received vehicle or AICAR (500 μg/g) during weeks 4–12 of feeding. Glucose tolerance was tested at week 11 by an IPGTT in (**a**) wild-type (*n* = 4 for all groups) and (**b**) *Adipoq* ^−/−^ (*n* = 3 for SFD and HFD, *n* = 7 for HFD + AICAR) mice. Circles, SFD; squares, HFD; triangles, HFD + AICAR. (**c**) Graph of the AUC of IPGTT curves. Data are presented as mean ± SEM. *p < 0.05, **p < 0.01, ***p < 0.001, ANOVA with Bonferroni correction; ^†^ *p* < 0.05, ^††^ *p* < 0.01, *Adipoq* ^−/−^ vs wild-type mice for respective conditions

An IPGTT was performed to assess glucose tolerance (Fig. 3a–c). HFD significantly impaired glucose clearance in both wild-type and *Adipoq* ^−/−^ mice (*p* < 0.001). However, HFD-fed *Adipoq* ^−/−^mice presented with exaggerated glucose intolerance compared with HFD wild-type controls (*p* < 0.05) (Fig. 3c).

AICAR did not significantly alter the HFD-induced increase in fasting blood glucose in either mouse strain (Fig. 3a,b). However, AICAR partially restored HFD-induced impairment of glucose clearance in wild-type mice (*p* < 0.05). This AICAR-mediated beneficial effect on glucose clearance was sustained in the *Adipoq* ^*−/−*^ mice (*p* < 0.05; Fig. 3c) and significantly lower levels of blood glucose were observed in AICAR-treated vs untreated HFD *Adipoq* ^*−/−*^at 60 min and 120 min post-glucose injection (Fig. 3b).

### AICAR attenuated HFD-induced hepatic steatosis independent of adiponectin

**Fig. 4:**
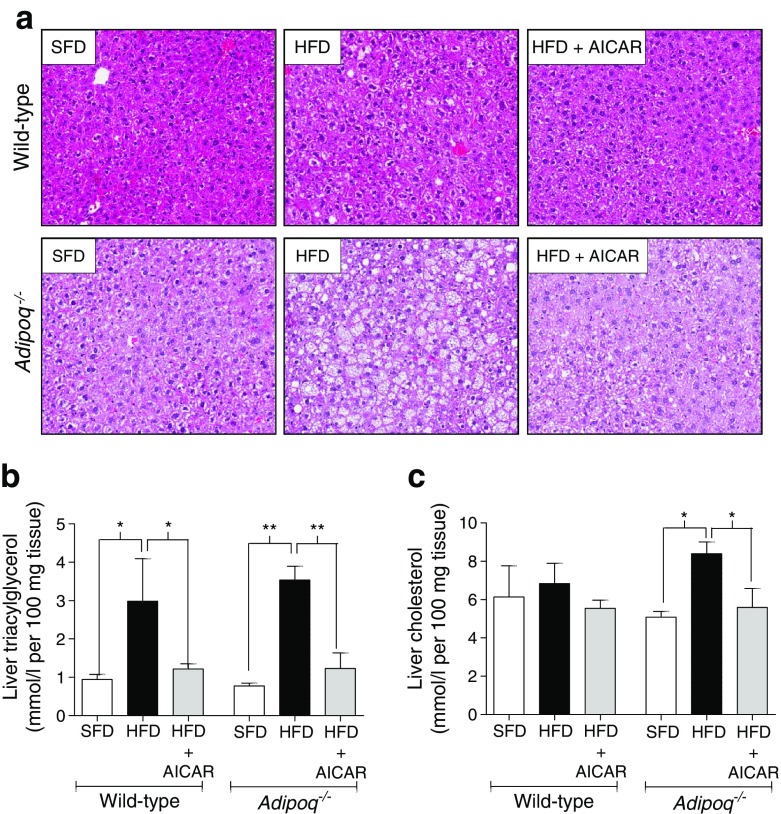
AICAR attenuates hepatic steatosis independent of adiponectin in obese mice. Wild-type and *Adipoq* ^−/−^ mice fed a 12 week SFD (10% fat) or HFD (60% fat) received vehicle or AICAR (500 μg/g) during weeks 4–12. (**a**) Representative images of hepatic haematoxylin and eosin staining (magnification × 40). Hepatic (**b**) triacylglycerol and (**c**) cholesterol (*n* = 3). Data are presented as mean ± SEM. *p < 0.05, **p < 0.01, ANOVA with Bonferroni correction

AICAR reduced HFD-induced hepatic steatosis, as evidenced by reduced hepatic vacuolisation (Fig. 4a) and triacylglycerol content (Fig. 4b). The drug also attenuated HFD-induced elevations in hepatic cholesterol levels in *Adipoq* ^−/−^ mice (*p* < 0.05; Fig. 4c). Furthermore, AICAR-mediated attenuation of hepatic vacuolisation was more pronounced in *Adipoq* ^−/−^ mice (Fig. 4a). Liver weight/hypertrophy was not altered by AICAR treatment (ESM Fig. 2).

### AICAR attenuated HFD-induced kidney disease independent of adiponectin

**Fig. 5:**
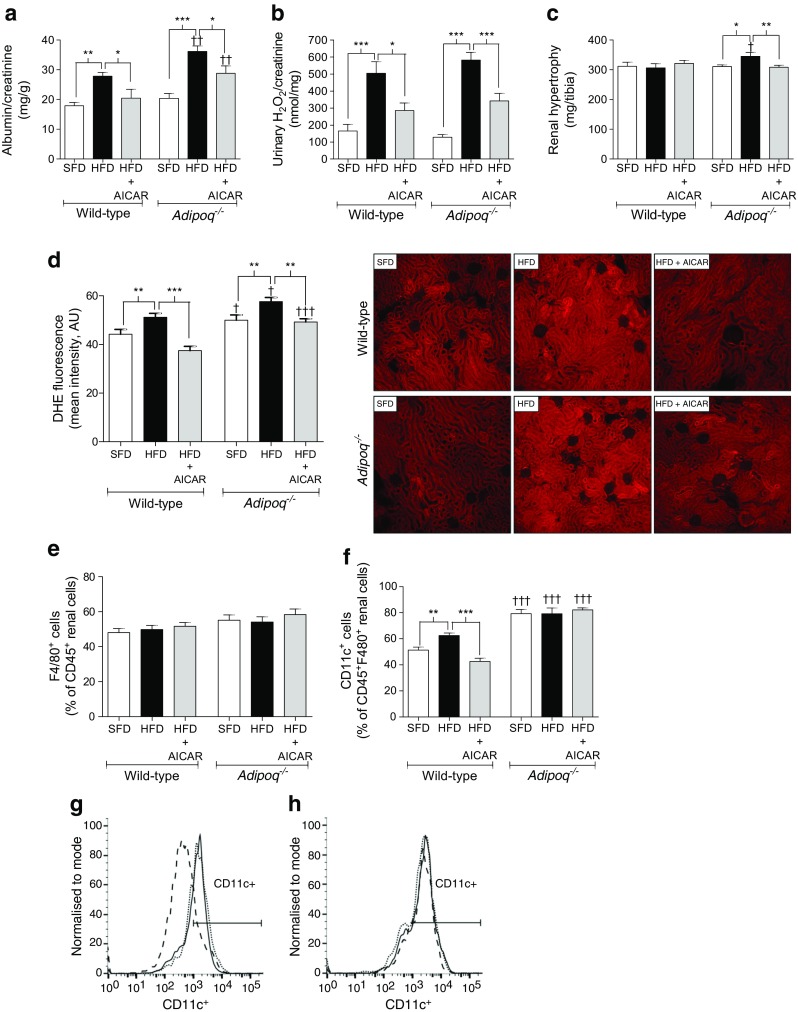
AICAR attenuates kidney disease independent of adiponectin in obese mice. Wild-type and *Adipoq* ^−/−^ mice fed a 12 week SFD (10% fat) or HFD (60% fat) received vehicle or AICAR (500 μg/g) during weeks 4–12. (**a**) Micro-albuminuria, (**b**) urine H_2_O_2_ and (**c**) renal hypertrophy were assessed (*n* = 7). (**d**) DHE was injected 16 h prior to killing the mice and renal DHE oxidation was quantified as a measurement of superoxide production (magnification × 100; *n* = 4). (**e**) Renal pan-macrophages (F4/80^+^) and (**f)** proinflammatory M1 macrophages (CD11c^+^) were characterised and quantified by flow cytometry (*n* = 5). (**g–h**) Flow cytometry histogram showing number of CD11c^+^ positive cells in (**g**) wild-type and (**h**) *Adipoq* ^−/−^ mice, incubated with vehicle (dotted line), fed an HFD without AICAR (solid line), or fed an HFD with AICAR (dashed line). Data are presented as mean ± SEM. *p < 0.05, **p < 0.01, ***p < 0.001, ANOVA with Bonferroni correction; ^†^ *p* < 0.05, ^††^ *p* < 0.01, ^†††^ *p* < 0.001, *Adipoq* ^−/−^ vs wild-type mice for respective conditions

Wild-type and *Adipoq* ^−/−^ mice developed significant renal dysfunction following a 3 month HFD regimen, as evidenced by increased albuminuria, urine H_2_O_2_ and renal superoxide production compared with SFD (Fig. 5a,b,d), without changes in plasma creatinine (ESM Fig. 3). Furthermore, renal hypertrophy was increased in HFD-fed *Adipoq* ^−/−^ mice compared with SFD (Fig. 5c). The total number of renal F4/80^+^ pan-macrophages was not altered by HFD in either mouse strain (Fig. 5e), but HFD significantly increased renal CD11c^+^ M1 macrophages in wild-type mice (Fig. 5f,g). Renal CD11c^+^ M1 macrophage levels were higher in *Adipoq* ^−/−^ mice compared with wild-type mice fed an SFD, but no additional increase was observed in HFD-fed *Adipoq* ^−/−^ mice (Fig. 5f,h).

AICAR significantly attenuated HFD-induced albuminuria (Fig. 5a), urinary H_2_O_2_ (Fig. 5b) and renal superoxide (Fig. 5d) in both wild-type and *Adipoq* ^−/−^ mice. Furthermore, AICAR attenuated HFD-induced renal hypertrophy in *Adipoq* ^−/−^ mice (Fig. 5c). Finally, AICAR completely attenuated the HFD-induced increase in renal CD11c^+^ M1 macrophages in the wild-type strain (Fig. 5f).

### AICAR reduced adipose inflammation in tissue explants obtained from obese human patients

WAT inflammation is a key driver of obesity-related pathophysiology [26–29]. As a proof-of-principle for translating our rodent data to human pathophysiology, we investigated whether AICAR could reduce inflammation and manipulate leucocyte phenotypes in omental WAT explants taken from obese patients undergoing gastric bypass surgery.

The antigens used to phenotype human macrophages varied slightly from the panel used for mice. Thus, we characterised human macrophages using the following activation markers: CD11c^+^ (M1), CD86^+^ (M1/M2b), CD206^+^ (M2a) or CD163^+^ (M2a/M2c). This classification is based on the Martinez et al scheme, whereby M1 macrophages display potent inflammatory activities, whereas M2a and M2c macrophages downregulate proinflammatory cytokines and promote resolution. Meanwhile, M2b macrophages produce IL-12 and IL-10, and are not anti-inflammatory per se, but rather activate the adaptive B cell responses and regulate B cell and T cell trafficking [33].

**Fig. 6:**
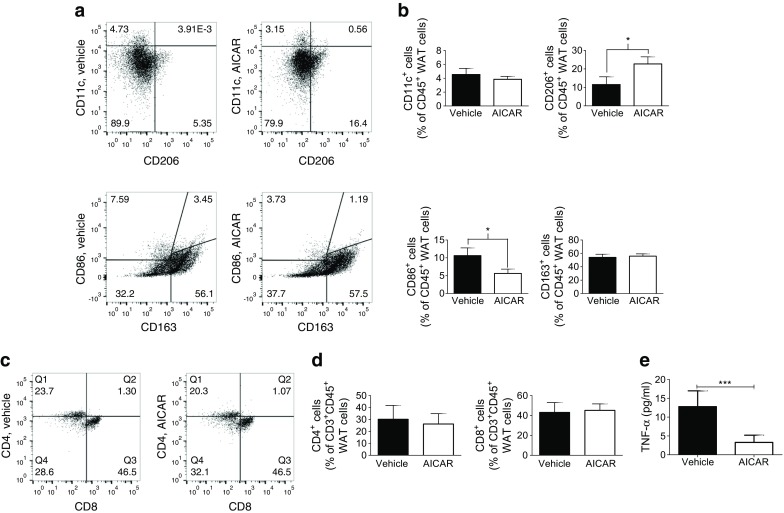
AICAR reduces inflammation in adipose explants from obese individuals. Omental WAT explants from obese individuals (BMI 35–50 kg/m^2^; *n* = 4 individuals) were incubated with vehicle or AICAR (1 mmol/l) for 6 h. (**a**) Tissue macrophages were characterised as M1 (CD11c^+^), M1/M2b (CD86^+^), M2a (CD206^+^) or M2a/M2c (CD163^+^) and (**b**) quantified. (**c**) T cells were characterised as CD4^+^ and CD8^+^ and (**d**) quantified. (**e**) Levels of TNF-α in the supernatant fraction were determined by ELISA. Data are presented as mean ± SEM. *p < 0.05, ***p < 0.001, Student’s *t* tests

Similar to our findings in mice, compared to vehicle AICAR promoted a shift towards inflammatory resolution in human WAT by increasing the percentage of anti-inflammatory CD206^+^ macrophages (*p* < 0.05), while reducing the percentage of proinflammatory CD86^+^ macrophages (*p* < 0.05). However, AICAR did not affect human CD11c^+^ or CD163^+^ macrophage expression (Fig. 6a,b), or the CD8^+^ or CD4^+^ T cell populations (Fig. 6c,d) in this 6 h ex vivo experiment. However, AICAR did reduce TNF-α secretion compared to vehicle (*p* < 0.001; Fig. 6e).

## Discussion

**Fig. 7:**
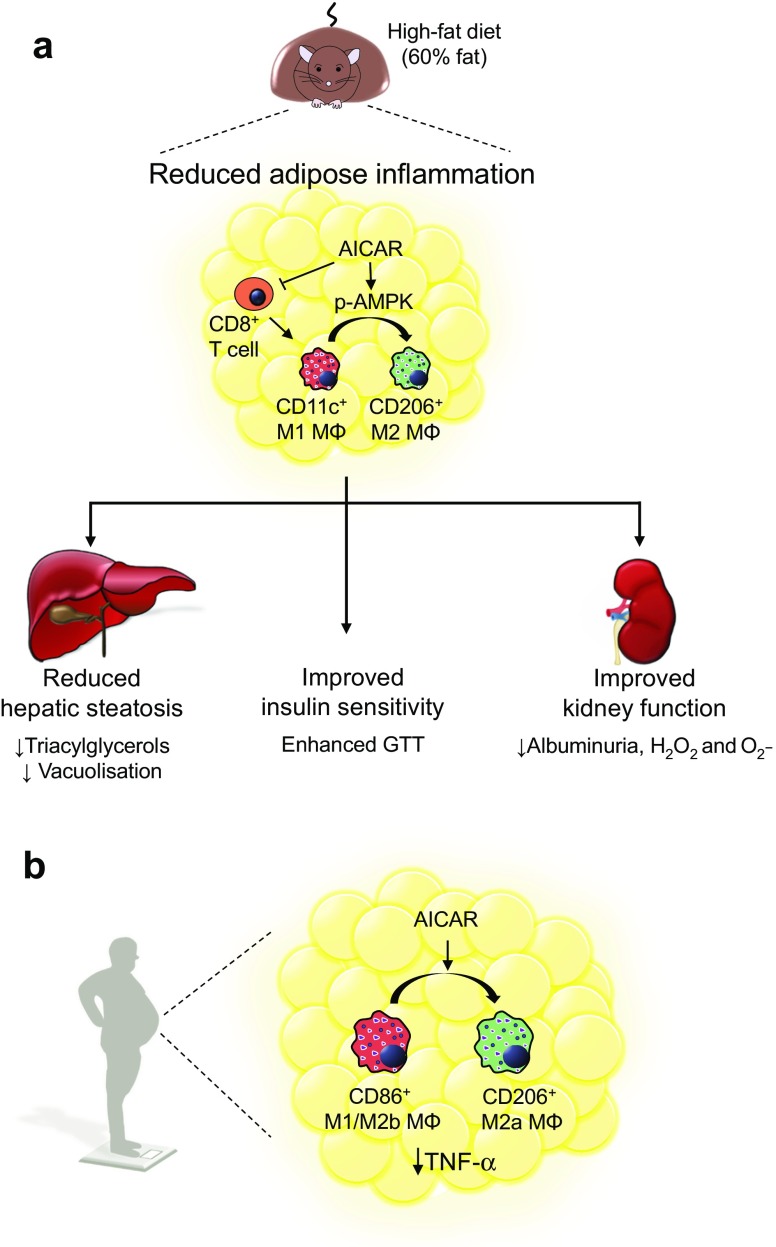
Schematic illustration of proposed AICAR-mediated effects in obesity. (**a**) In obese mice, AICAR treatment attenuates HFD-induced adipose inflammation, promoting an M1-to-M2 macrophage phenotype switch by reducing CD8^+^ T cell infiltration, while increasing p-AMPK levels. This results in reduced liver and kidney disease and enhanced glucose tolerance. All of these effects are independent of adiponectin. (**b**) AICAR mediates a similar M1-to-M2 macrophage phenotype switch in adipose explants isolated from obese individuals undergoing bariatric surgery. MΦ, macrophage

Obesity is an independent risk factor for numerous pathologies, including diabetes and liver and kidney disease [1, 2]. As the prevalence of obesity is increasing worldwide [3], the search for effective treatments against obesity-related pathophysiology is ongoing. Here we demonstrate that the AMPK-activating drug AICAR has therapeutic potential in this context. AICAR attenuates HFD-induced WAT inflammation and pathophysiology associated with diabetes, and liver and kidney disease in an adiponectin-independent manner. Collectively, these findings support a therapeutic potential for AICAR in attenuating HFD-induced pathophysiology (summarised in Fig. 7).

AICAR has previously been reported to increase metabolism and weight loss [7], even in sedentary mice [8]. Thus, it is not surprising that AICAR-treated HFD-fed mice gained less weight during the last weeks of the diet regimen, compared with vehicle-treated HFD-fed control mice. This effect on weight gain may have mediated some of the observed beneficial effects of AICAR, but it is unlikely that this is the sole protective mechanism of this compound since AICAR-treated HFD-fed mice weighed significantly more than controls fed an SFD.

Our observation that AICAR attenuates WAT inflammation may indicate a key mechanism of action as obesity-induced adipose inflammation is known to promote systemic pathophysiology [26, 27, 29, 34]. Indeed, inflammatory M1 macrophages infiltrating the obese WAT produce proinflammatory mediators (TNF-α, IL-1β, IL-6), which are associated with the development of insulin resistance and the subsequent release of NEFA, leading to systemic lipotoxicity, with effects on the liver and kidney [26–28, 35]. AICAR treatment promoted an M1-to-M2 macrophage phenotype switch, reducing the percentage of HFD-induced CD11c^+^ M1 macrophages, while restoring the CD206^+^ M2 macrophage population. Furthermore, AICAR increased WAT AMPK activity, which has been shown to promote an IL-10- producing M2 macrophage phenotype [36–38]. In cultured macrophages, AICAR also promoted an M1-to-M2 phenotype switch and increased AMPK activation, suggesting that the drug may directly manipulate macrophage cell signalling and phenotypic responses. Additionally, AICAR treatment reduced HFD-induced CD8^+^ T cell infiltration, which may have contributed to the attenuated inflammation since CD8^+^ T cells facilitate WAT accumulation of inflammatory CD11c^+^ M1 macrophages [39].

Hepatic steatosis is associated with obesity and diabetes and enhances susceptibility to liver disease [40, 41]. AICAR inhibited hepatic steatosis, reducing HFD-induced hepatic triacylglycerol accumulation in both wild-type and *Adipoq* ^**−/−**^ mice. This is in agreement with earlier studies demonstrating that AICAR reduces diet-induced hepatic triacylglycerol content in rats [9] and TNF-α-induced intracellular triacylglycerol accumulation in human hepatic HepG2 cell lines [11]. AICAR also attenuated HFD-induced hepatic cholesterol accumulation in *Adipoq* ^**−/−**^ mice, an interesting finding since AICAR-induced activation of AMPK inhibits the hepatic thyroid stimulating hormone (TSH)/sterol regulatory element-binding protein-2 (SREBP-2)/3-hydroxy-3-methylglutaryl-CoA reductase (HMGCR) pathway necessary for cholesterol biosynthesis [10].

Obesity is an independent risk factor for kidney disease and 25–40% of diabetic individuals develop nephropathy, which is the primary cause of end-stage renal failure [1, 2, 18]. In this study, we demonstrate that AICAR treatment attenuates cardinal features of HFD-induced kidney disease, namely microalbuminuria, production of reactive oxygen species and renal inflammation. AICAR did not affect the total number of renal macrophages in the mouse model used, but rather shifted their phenotype towards resolution by reducing the percentage of CD11c^+^ M1 macrophages. Collectively, this supports previous work from our group and others, demonstrating that AICAR attenuates both HFD-induced [12, 13] and diabetes-induced [14, 15] kidney disease. Importantly, in this study, we now also demonstrate that AICAR-mediated protection against kidney disease is independent of adiponectin. This is critical to the clinical application of AICAR as a potential therapeutic agent targeting kidney disease. Indeed, it has been debated whether the use of AICAR could be compromised in patients with diabetic nephropathy because research indicates that the drug may increase adiponectin [13]. Although adiponectin is generally described as a protective adipokine [16] and thought to protect podocyte function in early onset kidney disease [18, 42–44], the role of adiponectin in the later stages of human renal failure is unclear [18] with some studies suggesting that it is harmful [19, 20]. Thus, our finding that AICAR attenuated HFD-induced kidney disease in an adiponectin-independent manner may indicate that the drug is a more suitable therapeutic agent for patients with advanced nephropathy.

Importantly, *Adipoq* ^**−/−**^ mice presented with increased inflammation, liver vacuolisation and kidney injury compared with wild-type mice, probably because of the lack of the protective hormone adiponectin. Despite increased injury in the *Adipoq* ^**−/−**^ strain, AICAR maintained protection against WAT inflammation and liver and kidney injury. Thus, we conclude that this AICAR-mediated protection is independent of adiponectin. However, although AICAR maintained renoprotective effects in *Adipoq* ^**−/−**^ mice, it did not reduce the increased levels of renal CD11c^+^ M1 macrophages observed in these mice. Thus, these data indicate that AICAR-mediated renal protection is not mediated via reduced renal inflammation per se, but rather we hypothesise that the protection derives from the reduction of adipose inflammation, as illustrated in Fig. 7.

To translate our rodent data to human pathophysiology, we investigated if AICAR could reduce WAT inflammation in humans. AICAR promoted an M1-to-M2 macrophage phenotype shift in human WAT explants obtained from obese individuals. However, we observed important differences in the specific macrophage phenotypes affected in mice vs humans. AICAR did not affect M1 CD11c^+^ macrophage expression in human WAT, although M1/M2b CD86^+^ macrophage expression was reduced. This may be because AICAR did not affect the number of human CD8^+^ T cells, which drive CD11c^+^ macrophage infiltration [39]. Importantly, the ex vivo culture of human tissue with AICAR was limited to 6 h; thus it is possible that continuous therapeutic administration of the drug to patients may promote more substantial modulation of T cell and macrophage phenotypes. AICAR acted in a pro-resolving manner by increasing the anti-inflammatory CD206^+^ macrophage population in human WAT. Since CD163^+^ macrophage expression remained unaffected, AICAR may specifically promote the M2a phenotype. Finally, AICAR attenuated the level of TNF-α in human WAT, which is a key functional response in promoting metabolic health.

Collectively, these data support the use of AICAR to promote metabolic health and to protect against obesity-induced pathophysiology, such as liver steatosis and kidney disease. WAT inflammation is a common denominator of obesity-related pathologies, causing systemic lipotoxicity, insulin resistance and organ dysfunction. Thus, it is noteworthy that AICAR reduces WAT inflammation in both mice and humans. Importantly, AICAR protects against disease in an adiponectin-independent manner, which may make AICAR a suitable therapy for individuals with nephropathy.

## Electronic supplementary material

Below is the link to the electronic supplementary material.

ESM (PDF 419 kb)

## Abbreviations

AICAR: 5-Aminoimidazole-4-carboxamide ribonucleotide
AMPK: AMP-dependent protein kinase
CKD: Chronic kidney disease
DHE: Dihydroethidium
HFD: High-fat diet
SFD: Standard-fat diet
WAT: White adipose tissue
