# Supplementary material for: AICAR ameliorates high-fat diet-associated pathophysiology in mouse and ex vivo models, independent of adiponectin
Source: Diabetologia. 2017 Feb 10;60(4):729–39. doi: 10.1007/s00125-017-4211-9 (PMC6518112; doi:10.1007/s00125-017-4211-9)
Supplement: Supplementary file 1 — (PDF 419 kb) [file 125_2017_4211_MOESM1_ESM.pdf]

Electronically supplemental material (ESM) methods

Animal study

6-week-old male wild-type (#000664 C57BL/6J) and *Adipoq*$^{-/-}$ (#008195 B6;129-*Adipoq*^{tm1Chan}/J) mice were purchased from The Jackson Laboratory, Sacramento, CA, but were not littermates. Animals were fed a standard-fat-diet (SFD; 10% fat, Research Diets, Inc. Cat# D12450J) or a high-fat-diet (HFD; 60% fat; Research Diets, Inc., Cat# D12492) for 12 weeks. Diets contained equal protein content and matched sucrose content. Previous studies demonstrate that kidney disease is established after 4 weeks HFD-feeding [1]. Thus, as an interventional therapeutic, AICAR (500 μg/g) was given 3 times weekly via intraperitoneal (i.p.) injections, between weeks 4-12 (ESM Fig. 1a). Animals tolerated the treatment well, but displayed signs of hypothermia and cages were thus supplemented with heat 24 h post-injections. Animals were housed in a temperature/humidity controlled room on a 12 h light/12 h dark cycle and were allowed to acclimatize for a minimum of one week prior to experiments. All animal procedures were approved by the institutional animal care and use committee (IACUC), as detailed in the study approval section of the manuscript.

One week prior to sacrifice, a 24 h urine sample was collected through metabolic cages, where animals were housed in duplicate to minimise stress. Albumin, creatinine and albumin-to-creatinine ratio were analysed using DCA Vantage™ Analyzer (Siemens), to assess micro-albuminuria. Furthermore, a glucose-tolerance test (GTT) was performed the week prior to sacrifice. Briefly, mice were fasted for 4 h and D-glucose (1 mg glucose per gram body weight) was given intraperitoneally, after which tail-vein blood was drawn to measure plasma glucose levels using a Bayer glucometer at 0, 15, 30, 60 and 120 min post glucose challenge.

At time of harvest, organs were collected under isoflurane sedation to minimise hypoxia. Briefly, 500 mg perigonadal white adipose tissue (WAT) and one half of the right kidney (sectioned vertically) was immediately placed into PBS for flow cytometry analysis. The remaining tissues were either frozen in liquid nitrogen for isolation of protein or RNA, or fixed for a minimum of 24 h in 4% paraformaldehyde (PFA) and embedded in paraffin for immunohistochemistry analysis.

Liver function analysis

Liver tissue (100 mg) suspended in 3 mol/l KOH (in 65% EtOH) was incubated at 70°C for 1 h, to activate digestion, and diluted 1:3 with 2 mol/l Tris-HCl pH 7.5. Subsequently, total free cholesterol and triglycerides were determined in the liver extracts using standardised kits (WAKO Richmond, VA, Cat# 439-17501; Pointe Scientific, Canton, MI, Inc. Cat# T7532-500). Finally, paraformaldehyde fixed and paraffin embedded liver samples were stained by haematoxylin and eosin to visualise liver morphology and vacuolisation.

HPLC plasma creatinine analysis

Plasma (25 µl) was diluted 1:10 with ice-cold acidified acetonitrile (ACN), vortexed and placed on ice for 15 min. Following centrifugation (15 min, 15000 RPM, 4ºC), supernatants were collected and evaporated to dryness to remove the ACN, prior to reconstitution in 50 µl HPLC mobile phase (20 mmol/l sodium acetate pH = 4.2 ± 0.1). Samples were loaded onto a Perkin-Elmer HPLC autosampler tray and run at an isocratic load and elution at a flow rate of 0.3 ml/min over a Zorbax SCX Column (Agilent, Wilmington, DE: 150 mm x 2.1 mm, 5µ particle size) at 50 ± 0.5ºC and UV detector set at 225 nm, resulting in an elude time of approximately 3.5 min. Plasma

concentrations were determined by comparing eluted peaks to known creatinine standards (Cayman Biochemicals).

Renal superoxide measurements

Renal superoxide production was measured in a subgroup of mice (n=4 animals/group). These mice received intraperitoneal dihydroethidium (DHE) injections 16 h prior to harvest. Specifically, DHE (total 50 mg/kg) was administered via two injections of 25 mg/kg, given 30 min apart, in order to avoid the DMSO toxicity. At harvest, animals underwent cardiac perfusion for 1 min with saline, followed by 2 min perfusion with 4% paraformaldehyde (PFA) infusion. The kidneys were subsequently harvested and fixed for an additional 48 h in 4% PFA. Organs were sectioned using a vibratome into 55 µm slides and allowed to rest in PBS for 24 h before being mounted onto slides, in order to remove excess PFA and reduce nonspecific florescence. DHE oxidation in post-mortem slices were detected using confocal imaging, as a measurement of superoxide levels. Following imaging at 488 nM using a LSM 510 Laser Scanning Confocal Microscope, 12 individual Z-section stack (5x10 µm) were analysed per animals (n=4 animals/group).

Human adipose explant culture

Adipose biopsies were isolated from the omentum of obese (BMI 35-50 kg/m^2), non-diabetic bariatric surgery patients (n=4), undergoing laparoscopic antecolic-antegastric Roux-en-Y gastric bypass construction. Patients received pre-surgical antibiotics, but other medications (*e.g.* NSAIDs and anti-thrombotic drugs) were given after the collection of tissue biopsies. Larger microvasculature was dissected away and adipose explants were incubated *ex vivo* with vehicle or AICAR (1 mmol/l) for 6 h at 37˚C. 1 g tissue was used per 2 ml DMEM media. Supernatants were

collected and cleared of debris by centrifugation (500*g*, 5 min, 4°C) and TNF-α levels were determined using ELISA. Tissues were homogenised and leukocytes were isolated and characterised as described above.

Leukocyte phenotyping by flow cytometry

Adipose tissue was homogenised by 30 min collagenase treatment (5 mg/ml), with agitation at 37°C [3]. Kidneys were homogenised by 45 min treatment with 10 mg/ml collagenase, 10 mg/ml DNAse, 0.1 mmol/l β-mercaptoethanol, and 10% fetal-calf-serum, with agitation at 37°C [4]. Tissue lysates were filtered (70 μm) and underwent erythrocyte lysis. $5x10^5$ cells were stained by Aqua Live Dead (Thermo Fisher, Waltham, MA, Cat# L34957) and subsequently with relevant antibodies (ESM Table 1: mouse leukocytes, ESM Table 2: human leukocytes, purchased from eBioscience, San Diego, CA or BD Bioscience, San Jose, CA), for 20 min at room-temperature. In the case of murine leukocytes, cells were pre-gated as 'singlets', 'live cells', and leukocytes were identified as $CD45^+$. From this population, $F4/80^+$ MΦs were sub-categorised as inflammatory M1 MΦs ($CD11c^+$ of $CD45^+F480^+$ cells), *versus* M2 MΦs ($CD206^+$ of $CD45^+F480^+$ cells). T-cells were identified as $CD45^+CD3^+$cells and sub-characterised as $CD4^+$ *versus* $CD8a^+$. In the case of human leukocytes, lymphocytes and mononuclear/MΦ cell populations were identified by forward-versus-side scatter. Mononuclear/MΦ cells were characterised as M1 MΦs (% $CD11c^+$ of $CD45^+$ cells), versus M2a MΦs (% $CD206^+$ of $CD45^+$ cells) and M1/M2b MΦs (% $CD86^+$ of $CD45^+$ cells) versus M2a/c MΦs (% $CD163^+$ of $CD45^+$ cells). Lymphocytes were characterised as T-helper cells (% $CD4^+$ of $CD3^+CD45^+$ cells) and cytotoxic T- cells (% $CD8^+$ of $CD3^+CD45^+$ cells). All gating was determined using Fluorescence-Minus-One (FMO) controls and compensations were calculated using beads (eBioscience, San Diego, CA).

Western blot analysis

Protein lysate in RIPA buffer was obtained from homogenised adipose tissue and serum-starved J774 MΦs, which had been incubated with vehicle or AICAR (1 mmol/l) for 24 h. 40 µg protein was loaded onto a 16% SDS-PAGE gel and was transferred onto PVDF membranes (0.2 µm pore size) [2]. Proteins were identified using rabbit anti-p-AMPKα (Thr172) (no. 2535s), rabbit anti-AMPKα (no. 2532s) and rabbit anti-adiponectin (no. 2789) antibodies (all diluted 1:1000; Cell Signaling, Danvers, MA, USA). Proteins were normalised against β-actin (mouse anti-β-actin antibody [no. A2228]; Sigma, St Louis, MO, USA), diluted 1:10,000 and quantified using Adobe Photoshop. Original western blot images were cropped as indicated, to facilitate reader layout. Antibodies were validated by manufacturer.

References:

[1] Decleves AE, Mathew AV, Cunard R, Sharma K (2011) AMPK mediates the initiation of kidney disease induced by a high-fat diet. Journal of the American Society of Nephrology : JASN 22: 1846-1855

[2] Borgeson E, McGillicuddy FC, Harford KA, et al. (2012) Lipoxin A4 attenuates adipose inflammation. FASEB journal : official publication of the Federation of American Societies for Experimental Biology 26: 4287-4294

[3] Borgeson E, Johnson AM, Lee YS, et al. (2015) Lipoxin A Attenuates Obesity-Induced Adipose Inflammation and Associated Liver and Kidney Disease. Cell metabolism

[4] Teteris SA, Hochheiser K, Kurts C (2012) Isolation of functional dendritic cells from murine kidneys for immunological characterization. Nephrology 17: 364-371

ESM Table 1. Flow cytometry antibodies used to phenotype murine leukocytes.

**Mouse Macrophage & T-cell Panel**		
**Antibody**	**Product code**	**Company**
Anti-mouse CD19APC-eFluor® 780	47-0193-82	ebioscience
Anti-mouse CD3 eFlour 450	48-0032-82	ebioscience
Anti-mouse CD4 Alexa Fluor 700	56-0042-82	ebioscience
Anti-mouse CD8a PE-Cy7	25-0081-82	ebioscience
Anti-mouse CD11b APC	17-0112-82	ebioscience
Anti-mouse CD11c Per-CP cy5.5	45-0114-82	ebioscience
Anti-mouse F4/80 PE	12-4801-82	ebioscience
Anti-mouse CD206 FITC	MCA2235FT	AbDserotec

ESM Table 2. Flow cytometry antibodies used to phenotype human leukocytes.

**Macrophage Panel**		
**Antibody**	**Product code**	**Company**
Anti-Human CD11c Alexa Fluor® 700	56-0116-42	ebioscience
Anti-Human CD86 (B7-2) Alexa Fluor® 488	53-0869-41	ebioscience
Anti-Human CD206 (MMR) eFluor® 450	48-2069-41	ebioscience
Anti-Human CD68 PE-Cyanine7	25-0689-41	ebioscience
Anti-Human CD197 (CCR7) APC-eFluor® 780	47-1979-41	ebioscience
Anti-Human CD163 PerCP-eFluor® 710	46-1639-41	ebioscience
Anti-Human CD45 APC	840910	BD Biosciences
**T-cell Panel**		
**Antibody**	**Product code**	**Company**
Anti-Human CD3 Pac Blue	558124	BD Biosciences
Anti-Human CD8 PerCP	347314	BD Biosciences
Anti-Human CD4 AlexaFlour700	557922	BD Biosciences
Anti-Human CD45 APC-H7	560178	BD Biosciences

ESM Fig. 1

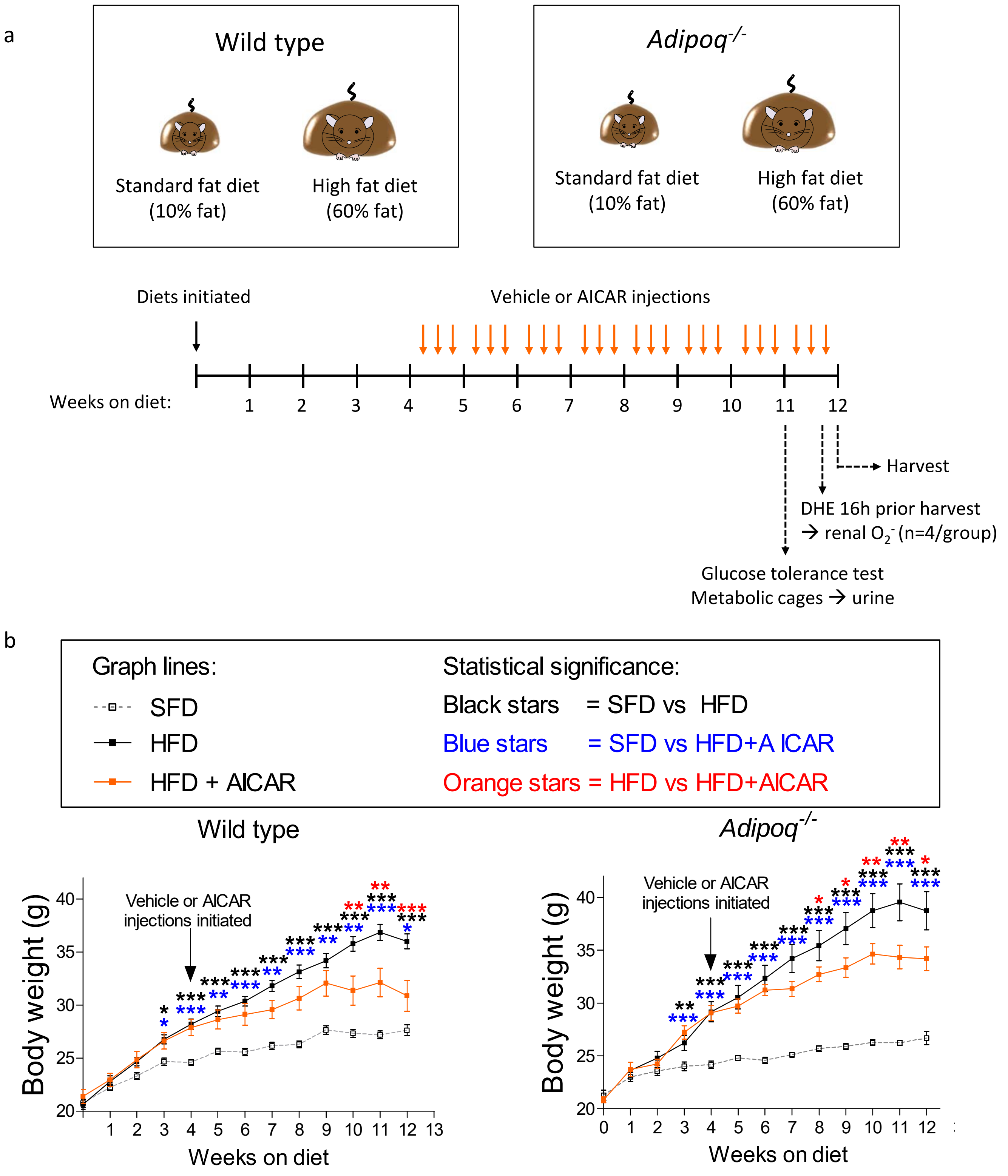

ESM Fig 1. Schematic illustration of mouse study and animal weight gain.
a) Wild-type and *Adipoq*$^{-/-}$ mice fed a 12-week standard-fat diet (SFD; 10% fat) or high-fat diet (HFD; 60% fat) received vehicle or AICAR (500 µg/g) between weeks 4-12. b) Body weight was recorded weekly (n=17). Data are presented as mean±SEM and were analysed by ANOVA with Bonferroni correction, **p*<0.05, ***p*<0.01, ****p*<0.001.

ESM Fig. 2

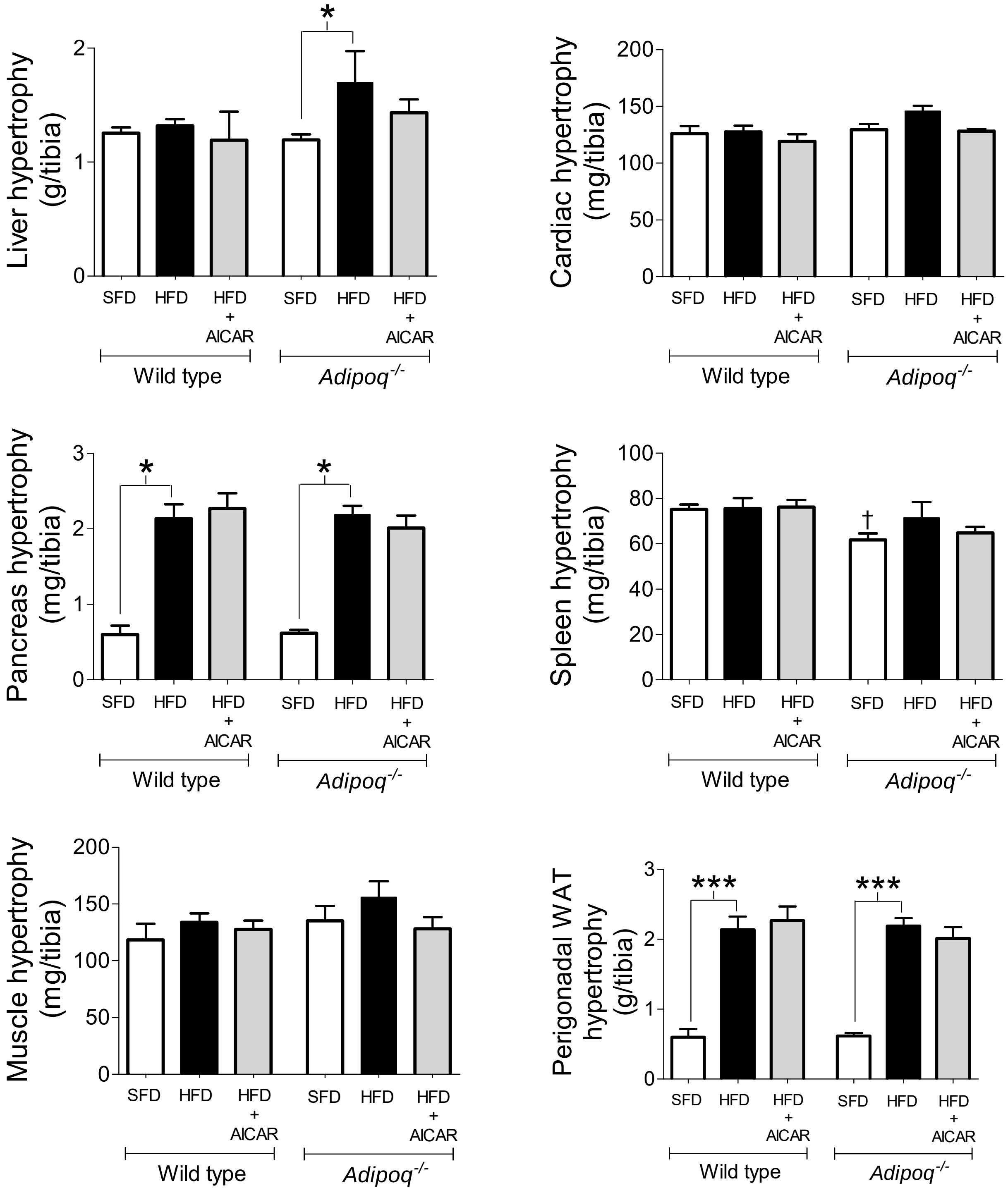

ESM Fig 2. Organ hypertrophy.

Wild-type and *Adipoq*$^{-/-}$ mice fed a 12-week standard-fat diet (SFD; 10% fat) or high-fat diet (HFD; 60% fat) received vehicle or AICAR (500 µg/g) between weeks 4-12. Organ hypertrophy was recorded and normalised to the length of the tibia, as indicated, n=7-10. Data are presented as mean±SEM and were analysed by ANOVA with Bonferroni correction, **p*<0.05, ****p*<0.001.

ESM Fig. 3

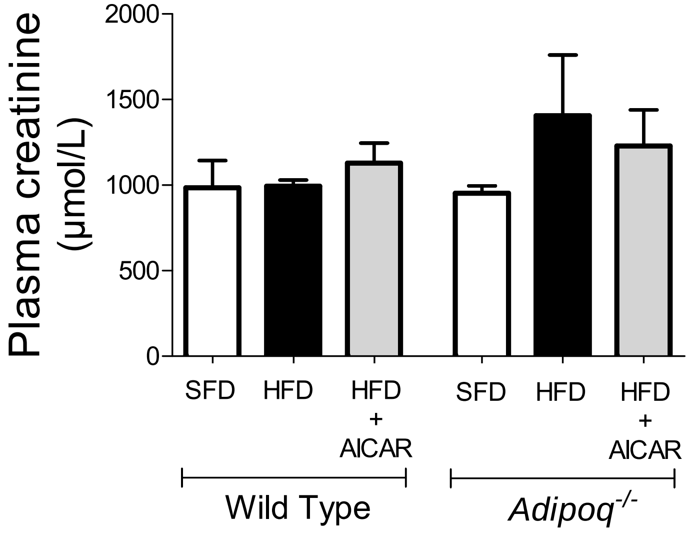

ESM Fig 3. Effects of HFD and AICAR on plasma creatinine in obese mice.
Wild-type and *Adipoq*$^{-/-}$ mice fed a 12-week standard-fat diet (SFD; 10% fat) or high-fat diet (HFD; 60% fat) received vehicle or AICAR (500 µg/g) between weeks 4-12. Blood was collected via a mandibular blood draw and plasma creatinine was determined by HPLC (n=10). Data are presented as means ± SEM and analysed by ANOVA with Bonferroni correction.

ESM Fig. 4

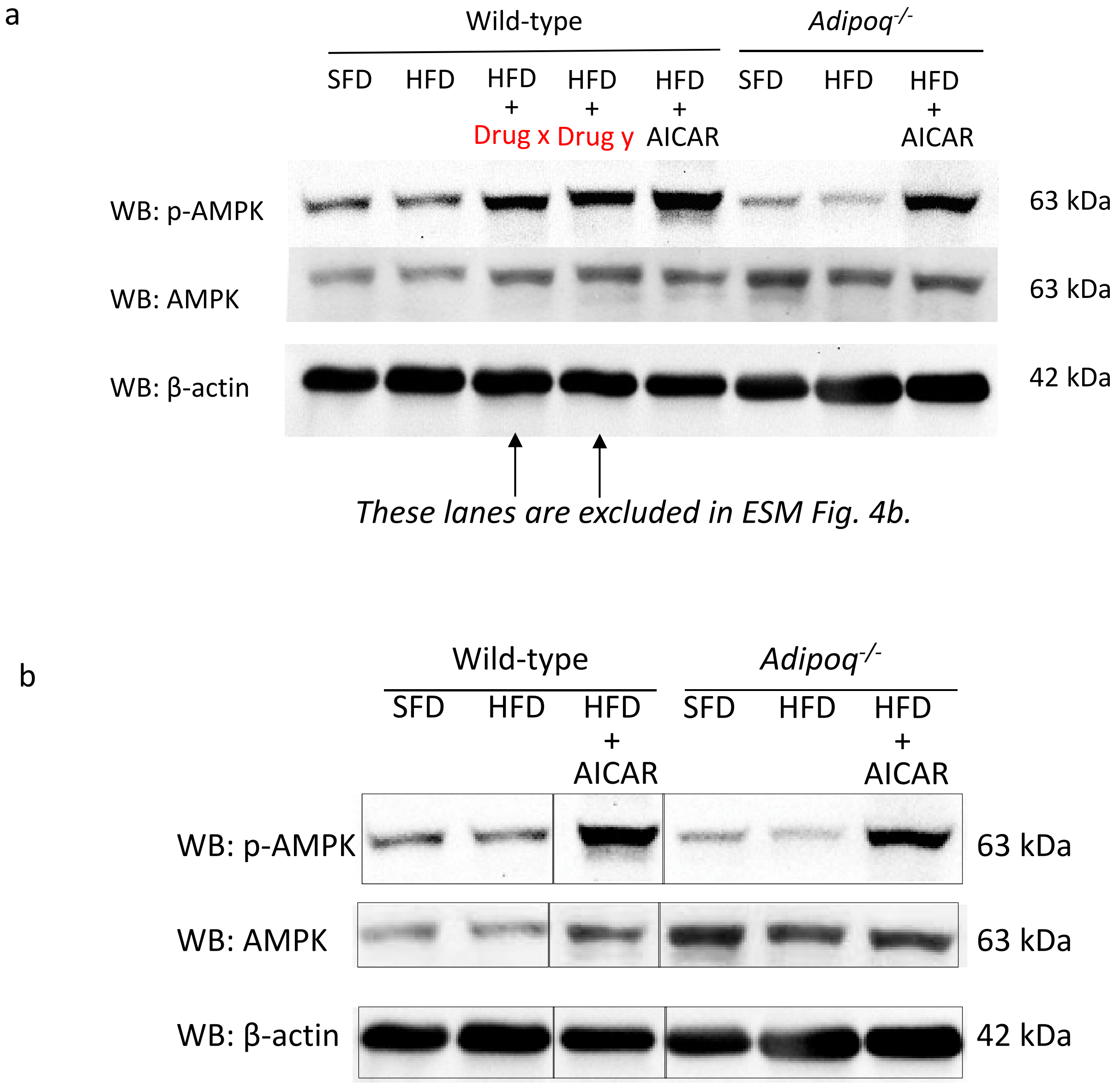

ESM Fig 4. Western blots.
Wild-type and *Adipoq*$^{-/-}$ mice fed a 12-week standard-fat-diet (SFD; 10% fat) or high-fat-diet (HFD; 60% fat) received vehicle or AICAR (500 µg/g) between weeks 4-12. Adipose tissue was homogenised and p-AMPK/AMPK activity was assessed by western blot. a) Original western blots. b) Cropped western blots, where lanes 3 and 4 (from the left) were excluded to facilitate reader layout. This cropped western blot is presented in Fig. 1i of the main manuscript.
